# How the *Community Eye Health Journal* supports learning in Zambia and Ghana

**Published:** 2024-02-09

**Authors:** Juliet Mulenga, Louis Oteng-Gyimah

**Affiliations:** 1 Ophthalmologist: University Teaching Hospitals, Lusaka, Zambia.; 2 Ophthalmologist: Anglican Eye Clinic, Jachie, Ghana

**Our supporters show how eye care professionals are using the *Community Eye Health Journal* in their place of work.**

**Juliet Mulenga** is an ophthalmologist at the University Teaching Hospitals – Eye Hospital in Lusaka, Zambia and **Louis Oteng-Gyimah** is an ophthalmologist at the Anglican Eye Clinic in Jachie, Ghana. They are both alumni of the MSc Public Health for Eye Care offered by the International Centre for Eye Health at the London School of Hygiene & Tropical Medicine (http://tinyurl.com/ICEHmsc).

Last year, Juliet and Louis, who are both *Journal* readers, offered to help ensure that the *Community Eye Health Journal* reaches everyone in their country who needs it, in the most appropriate format – whether that is via our website (www.cehjournal.org), via our handy smartphone app (see panel), or as print copies.

These photos show how they, and their colleagues, use the *Community Eye Health Journal* in their daily work.

**Did you know?**

**Figure:**
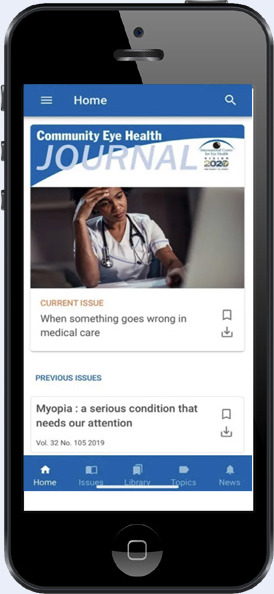

Our smartphone app has a Library feature that allows you to download articles to your phone and save them in your own set of named folders – ready for outreach, teaching, or talking to patients about their eye condition. You can access these articles even when you've run out of data!

**Figure:**
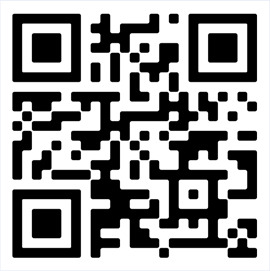

**We would love to hear from you!**

How is the *Community Eye Health Journal* used in your place of work? Send us your photos and stories!

Please write to: editor@lshtm.ac.uk

**Figure:**
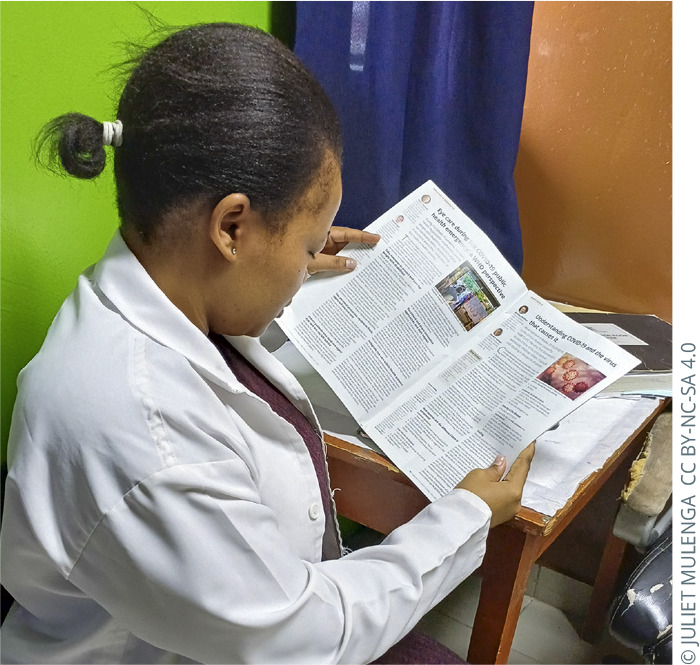
A second-year resident reads up in preparation for an eye care presentation. ZAMBIA

**Figure:**
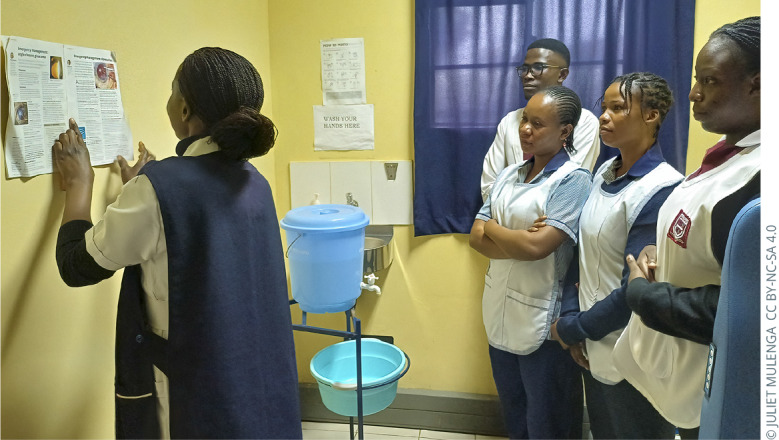
A senior nurse uses the eye emergency issue from 2018 (bit.ly/eye-emergencies) to teach student nurses about different emergency conditions and their management. zambia

**Figure:**
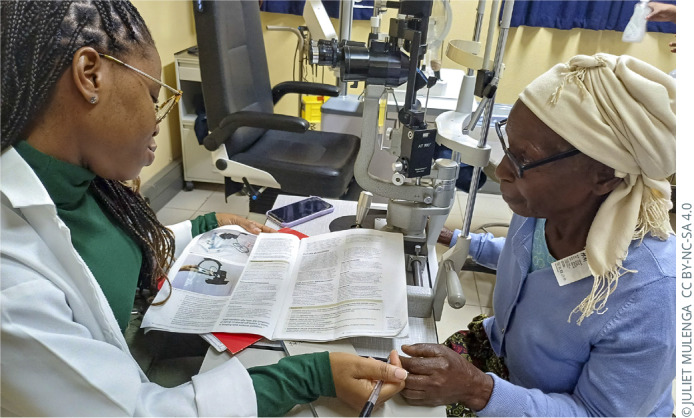
A resident ophthalmologist uses the journal to talk to a patient about their eye condition. zambia

**Figure:**
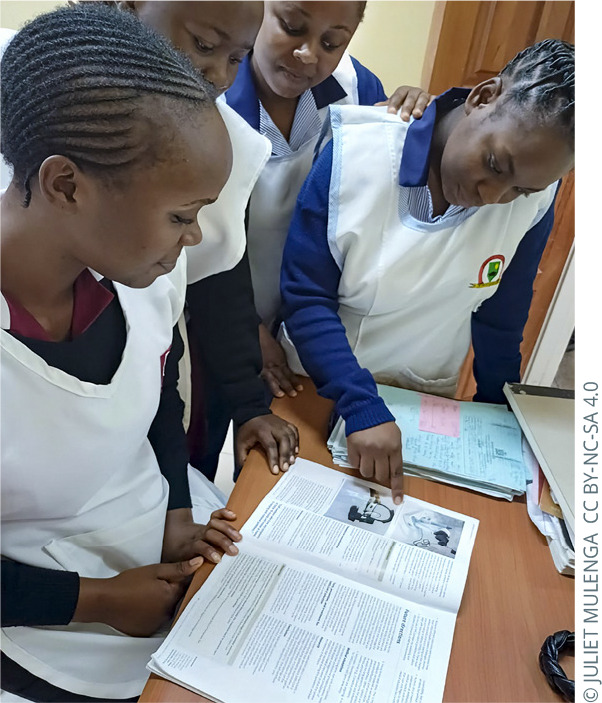
Student nurses learning how eye equipment can be modified to reduce the spread of infections. zambia

**Figure:**
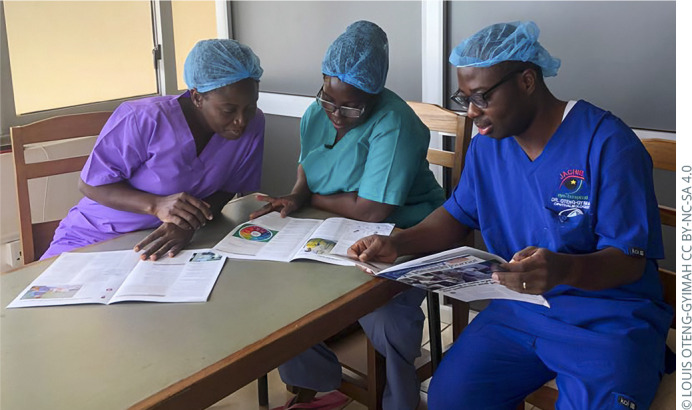
The surgical team discuss articles from the ‘Running a safe eye service issue (bit.ly/SafeEye). ghana

**Figure:**
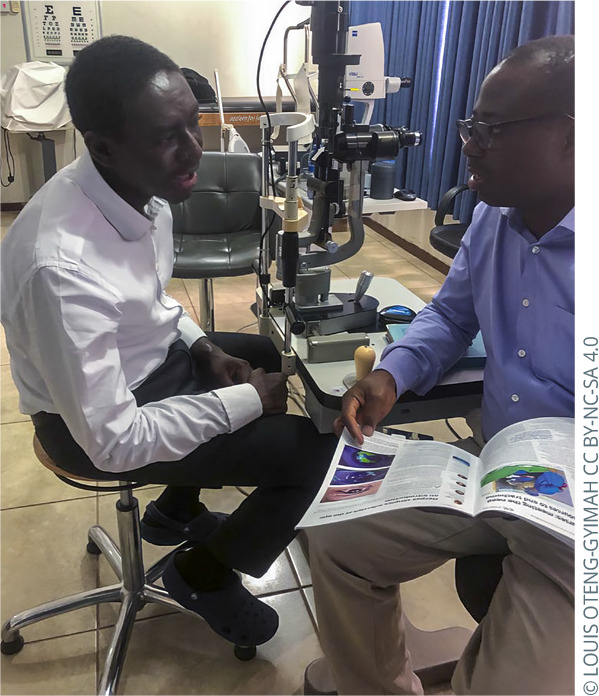
Explaining herpes simplex infection of the cornea to a client in the consulting room. ghana

